# NLRP3 Controls *Trypanosoma cruzi* Infection through a Caspase-1-Dependent IL-1R-Independent NO Production

**DOI:** 10.1371/journal.pntd.0002469

**Published:** 2013-10-03

**Authors:** Virginia M. Gonçalves, Kely C. Matteucci, Carina L. Buzzo, Bruna H. Miollo, Danny Ferrante, Ana C. Torrecilhas, Mauricio M. Rodrigues, Jose M. Alvarez, Karina R. Bortoluci

**Affiliations:** 1 Centro de Terapia Celular e Molecular (CTC-Mol), Universidade Federal de São Paulo, Vl Clementino, São Paulo, Brazil; 2 Departamento de Ciências Biológicas - ICAQF, Universidade Federal de São Paulo, São Paulo, Brazil; 3 Departamento de Microbiologia, Imunologia e Parasitologia, Universidade Federal de São Paulo, São Paulo, Brazil; 4 Departamento de Imunologia, Instituto de Ciências Biomédicas, Universidade de São Paulo, Cidade Universitária, São Paulo, Brazil; Harvard School of Public Health, United States of America

## Abstract

*Trypanosoma cruzi* (*T. cruzi*) is an intracellular protozoan parasite and the etiological agent of Chagas disease, a chronic infectious illness that affects millions of people worldwide. Although the role of TLR and Nod1 in the control of *T. cruzi* infection is well-established, the involvement of inflammasomes remains to be elucidated. Herein, we demonstrate for the first time that *T. cruzi* infection induces IL-1β production in an NLRP3- and caspase-1-dependent manner. Cathepsin B appears to be required for NLRP3 activation in response to infection with *T. cruzi*, as pharmacological inhibition of cathepsin B abrogates IL-1β secretion. NLRP3^−/−^ and caspase1^−/−^ mice exhibited high numbers of *T. cruzi* parasites, with a magnitude of peak parasitemia comparable to MyD88^−/−^ and iNOS^−/−^ mice (which are susceptible models for *T. cruzi* infection), indicating the involvement of NLRP3 inflammasome in the control of the acute phase of *T. cruzi* infection. Although the inflammatory cytokines IL-6 and IFN-γ were found in spleen cells from NLRP3^−/−^ and caspase1^−/−^ mice infected with *T. cruzi*, these mice exhibited severe defects in nitric oxide (NO) production and an impairment in macrophage-mediated parasite killing. Interestingly, neutralization of IL-1β and IL-18, and IL-1R genetic deficiency demonstrate that these cytokines have a minor effect on NO secretion and the capacity of macrophages to control *T. cruzi* infection. In contrast, inhibition of caspase-1 with z-YVAD-fmk abrogated NO production by WT and MyD88^−/−^ macrophages and rendered them as susceptible to *T. cruzi* infection as NLRP3^−/−^ and caspase-1^−/−^ macrophages. Taken together, our results demonstrate a role for the NLRP3 inflammasome in the control of *T. cruzi* infection and identify NLRP3-mediated, caspase-1-dependent and IL-1R-independent NO production as a novel effector mechanism for these innate receptors.

## Author Summary

Inflammasomes are cytosolic innate receptors that are emerging as central effectors in the control of infections and inflammatory pathologies. NLRP3 is the most studied member of inflammasomes with established role in the control of bacterial and viral infections. This manuscript describes original studies on the involvement of NLRP3 inflammasome in the control of *Trypanosoma cruzi*, the etiological agent of Chagas disease, a chronic infectious illness that affects millions of people in the world. *T. cruzi* activates NLRP3 inflammasome by a mechanism involving cathepsin B. NLRP3^−/−^ and caspase1^−/−^ mice display high parasitemia during acute phase of *T. cruzi* infection, which could be explained by a severe defect in the production of nitric oxide (NO) and in the impairment of their macrophages to control intracellular parasites. Interestingly, inhibition of caspase-1, but not the neutralization of IL-1β and IL-18, the best-studied caspase-1 substrates, abrogated NO production by WT and MyD88^−/−^ macrophages and rendered them as susceptible to *T. cruzi* infection as NLRP3^−/−^ macrophages. Together, our results indicate a caspase-1-dependent and IL-1β and IL-18-independent pathway for NO production as a new effector mechanism played by NLRP3 to control *T. cruzi* infection.

## Introduction

*Trypanosoma cruzi* is an intracellular trypanosomatid protozoan that is transmitted to the human host by blood-feeding *Reduviidae* bugs from the subfamily Triatominae. *T. cruzi* is the causative agent of Chagas disease and American trypanosomiasis, a chronic infectious disease. While Chagas disease is endemic in Latin America, a significant increase in confirmed cases of Chagas disease has recently been reported in the USA, Canada, Japan, Australia and Europe, indicating that it is an emerging disease [1] [2] [3]. Due to increasing immigration from endemic countries and a lack of regular screening in blood banks and hospitals (with a few exceptions), *T. cruzi* infection is a potential public health issue in the USA and Europe.

The control of *T. cruzi* by the immune system depends on both innate and adaptive responses. Innate immune cells are responsible for the initial recognition of the parasite, as well as the initiation and coordination of adaptive responses [4]. The transmembrane Toll-like Receptor (TLR) family of pattern recognition receptors (PRRs) plays a central role in the recognition of *T. cruzi* by the immune system [5]. TLR4 [6], TLR2 [7] [8] [9], TLR9 [10] and TLR7 [11] are responsible for sensing glycoinositolphospholipid-containing ceramides (GIPLs), glycosylphosphatidylinositol (GPI) anchors from the trypomastigote form of the parasite (t-GPI mucin), *T. cruzi* DNA and *T. cruzi* RNA, respectively. These receptors initiate a signaling cascade that is dependent on the adaptor molecule MyD88 and culminates in the activation of pro-inflammatory genes that are crucial for resistance to *T. cruzi* infection, including IL-12 [12] [13] [14] [15], IFN-γ [16] and the microbicidal molecule nitric oxide (NO) [17] [18]. MyD88^−/−^ mice are highly susceptible to *T. cruzi* infection, possibly because of defects in the production of pro-inflammatory cytokines [19]. In addition to TLR, NOD1, a member of the cytosolic NOD-like receptor (NLR) family, plays a role in controlling *T. cruzi* infection [20]. NOD1^−/−^ macrophages exhibit impaired production of pro-inflammatory cytokines and NO, and NOD1^−/−^ mice succumb to the acute phase of *T. cruzi* infection. Despite evidence for the critical role of NOD1 in controlling *T. cruzi*, the agonist responsible for NOD1 activation remains unknown.

In addition to NOD1 and NOD2, the cytosolic PRR family includes inflammasomes, which are multiprotein complexes that contain a sensor protein from the NLR or pyrin and HIN domain-containing protein (PYHIN) families. The signaling cascade initiated by inflammasomes includes the recruitment of the apoptosis-associated speck-like (ASC) protein (which contains a caspase activating and recruitment domain (CARD) and pro-caspase-1 or pro-caspase-11 (pro-caspase-4 in humans), culminating in the activation of these proteases (reviewed in [21] [22] [23]). Active caspase-1 mediates the maturation and secretion of the IL-1β and IL-18 pro-inflammatory cytokines and, along with caspase-11, induces the pyroptosis cell death program.

NLRP3, NLRC4 and AIM2 are the best-characterized inflammasome complexes. NLRC4 is activated in response to bacterial proteins such as flagellin [24] or the inner rod component of the bacterial type III secretion system (T3SS) [25], whereas AIM2 senses cytosolic bacterial [26] and viral DNA [27]. NLRP3 is activated by several different classes of molecules, including pathogen-associated molecular patterns (PAMPs) from viruses [28], bacteria [29] [30] [31], fungi [32] and protozoa [33]; signals from endogenous damage-associated molecular patterns (DAMPS) such as uric acid and ATP [34] [35]; and crystalline or particulate structures such as alum [36] [37] and silica [38]. NLRP3 activation by these molecules, which are not structurally related, appears to be influenced by ROS production, lysosomal rupture or potassium efflux, which are believed to result in distinct activation signals [39] [40] [41].

Inflammasomes have been implicated in the host response to several pathogens. NLRP3 has been identified as an effector in the control of *Mycobacterium* [42], *Cytrobacterium* [43], *Salmonella* [44], *Brucella* [45], Influenza A virus [46] [47] [48], *Candida* [32] and other pathogens. However, the *in vivo* role of the NLRP3 inflammasome in the host defense against *T. cruzi* has not been elucidated. Here, we demonstrate that the NLRP3 inflammasome controls *T. cruzi* parasitemia by inducing NO production via a caspase-1-dependent, IL-1R-independent pathway.

## Methods

### Ethics statement

This study was carried out in strict accordance with the recommendations in the Guide for the Care and Use of Laboratory Animals of the Brazilian National Council of Animal Experimentation (http://www.cobea.org.br/). The protocol was approved by the Committee on the Ethics of Animal Experiments of the Institutional Animal Care and Use Committee at the Federal University of Sao Paulo (Id # CEP 0162/11).

### Animals

C57BL/6 wild type (WT), caspase-1^−/−^, NLRP3^−/−^, MyD88^−/−^, IL-1R^−/−^, iNOS^−/−^ and IFN-γ^−/−^ mice were bred in our animal facilities at the Federal University of São Paulo. The caspase-1^−/−^ mice were kindly provided by Dr. Richard Flavell (Yale University, USA), and the NLRP3^−/−^ mice were kindly provided by Dr. Vishva Dixit (Genentech, USA). Eight-week-old female mice were infected subcutaneously (s.c.) with 10^3^ blood-derived trypomastigotes from the Y strain of *T. cruzi*, which was obtained from the Dante Pazzanese Institute. Parasitemia was monitored by counting the number of bloodstream trypomastigotes in 5 µL of fresh blood collected from the tail vein. Spleens were removed 10 days after infection. All animal procedures were developed with all efforts to minimize suffering and were approved by the Ethics Committee for Animal Care of the Federal University of São Paulo UNIFESP (Id # CEP 0162/11).

### Preparation of mouse macrophages

Peritoneal macrophages (PMs) were obtained by peritoneal lavage 4 days after intraperitoneal injection of a 1% starch solution (Sigma Aldrich). Peritoneal cells were cultured overnight in RPMI 1640 medium supplemented with 3% heat-inactivated FCS and antibiotics. Non-adherent cells were removed by washing with warm RPMI medium.

### In vitro infection with *T. cruzi*

PMs (1,5×10^6^ cells/300 µl) cultured in RPMI medium were infected with trypomastigotes from *T. cruzi* strain Y (3 parasites/cell) purified from a monkey epithelial cells (LLCMK_2_). The cells were treated with a caspase-1 inhibitor (z-YVAD-fmk) (10 µM) (MBL International Corporation), an iNOS inhibitor (1 mM) (Aminoguanidin, AG), a cathepsin B inhibitor (Ca-074-Me) (Sigma Aldrich), a K+ channel inhibitor (Glybenclamide, GLB) (InVivogen), an ROS inhibitor (Apocynin, APO) (25 µM to 100 µM) (Sigma Aldrich) or neutralizing antibodies against IL-1β and IL-18 (0,1, 1 and 10 ng/mL) (BD Pharmingen). Supernatants were collected 24 h after infection to detect IL-1β, IFN-γ and IL-6, and 48 h after infection to measure the NO concentration.

### Microbicidal activity of macrophages

PMs (3×10^5^) from C57BL/6 wild type (WT), caspase-1^−/−^, NLRP3^−/−^, MyD88^−/−^, iNOS^−/−^ and IL-1R^−/−^, mice were infected with trypomastigotes from *T. cruzi* Y (1∶5) for 2–4 h. Extracellular parasites were removed by washing, and after 48 hours the chambers were fixed with methanol and stained with DAPI (4′, 6-Diamidino-2-phenylindole) (Sigma Aldrich). The frequency of infected macrophages and the number of amastigotes inside the macrophages were evaluated by fluorescence microscopy (600×). At least 500 macrophages were counted for each representative experiment.

### Measurement of cytokines and NO

Supernatants from *in vitro*-infected PMs (1,5×10^6^ cells/300 µl) or cultured spleen cells from infected mice (3×10^6^ cells/300 µl) were collected after 24 h. IL-1β and IL-6 were measured using enzyme-linked immunosorbent assay (ELISA) kits from BD Pharmingen (OptEIA) according to the manufacturer's instructions. The levels of IL-1β were determined using a standard curve generated with the 17 KDa mature form of recombinant mouse IL-1β, as previously described [49]. IFN-γ was detected by sandwich ELISA using a purified anti-IFN-γ capture antibody (BD Pharmingen) and a biotin rat anti-mouse IFN-γ antibody (BD Pharmingen). IFN-γ levels were calculated based on a standard curve generated using recombinant cytokine (BD Pharmingen). The nitrite concentration was determined by the Griess reaction. Briefly, 50 µl of Griess reagent solution (1% sulfanilamide, 0.1% naphthylene diamine dihydrochloride, 2% H_3_PO_4_) was added to 50 µl samples, and the absorbances were measured at 540 nm.

### Statistical analysis

Experiments were performed in duplicate or triplicate, and at least three independent experiments were performed. Data are presented as the mean ± S.D. Statistical analysis of the data was carried out using one-way or two-way ANOVA and Tukey's post-test. Differences between the experimental groups were considered significant as follows: *p*<0.05 (*), *p*<0.01 (**) and *p*<0,001(***).

## Results

### *T. cruzi*-induced NLRP3 activation is dependent on cathepsin B

**Figure 1:**
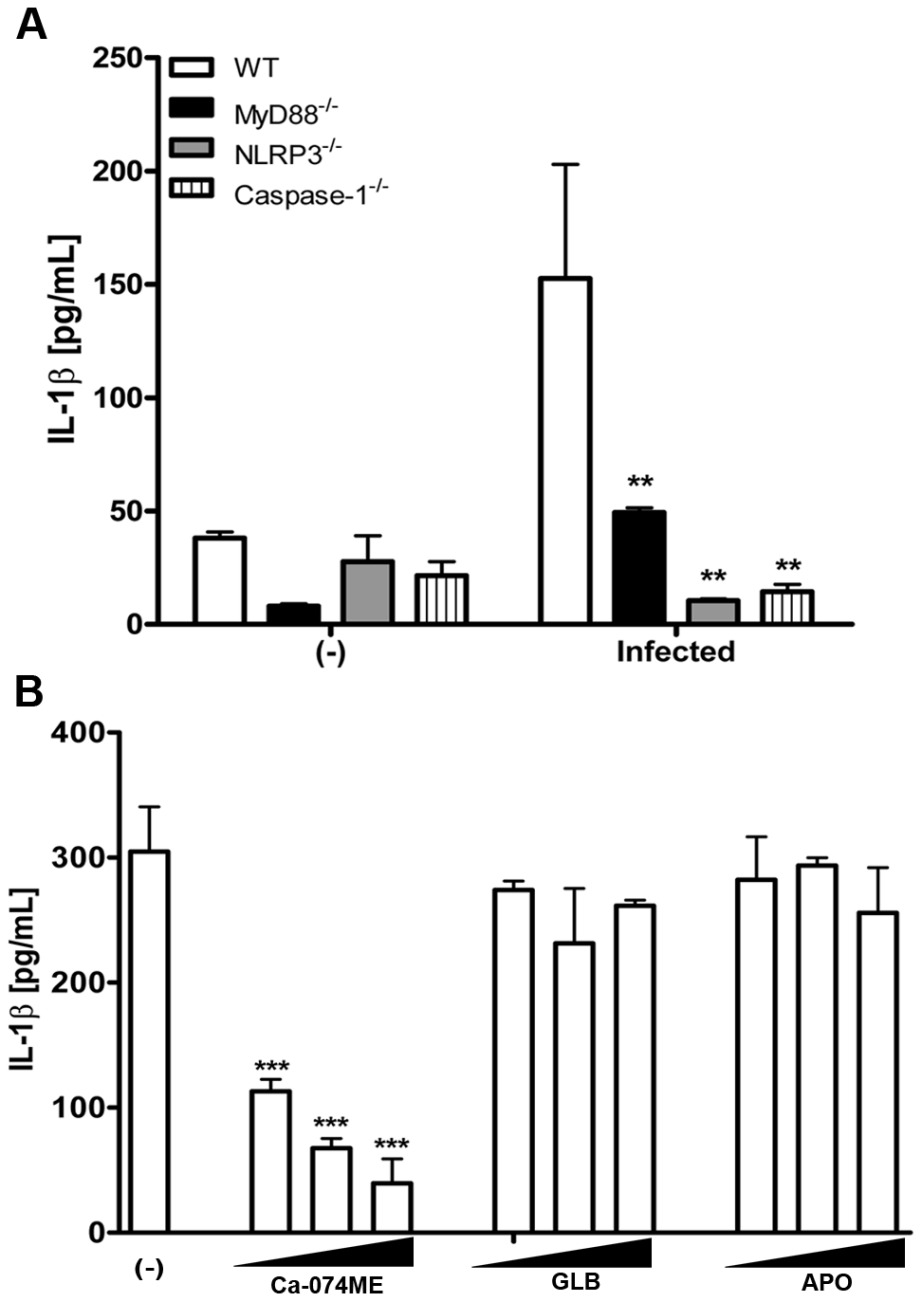
*T. cruzi*-induced NLRP3 activation. PMs from WT, NLRP3^−/−^ and caspase-1^−/−^ mice (1,5×10^6^ cells/300 µl) were infected with *T. cruzi* Y trypomastigotes (3 parasites per cell). The supernatants were collected after 24 h for the evaluation of IL-1β levels by ELISA. Bars represent the mean ± S.D. of triplicate samples, where ** p>0,01 compared to the WT group (**A**). *T. cruzi*-infected PMs (3 parasites per cell) (1,5×10^6^ cells/300 µl) were treated with Ca-074ME, APO or GLB in doses of 25, 50 and 100 µM, or left untreated (-). After 24 h, the supernatants were collected and IL-1β production was evaluated by ELISA. Bars represent the mean ± S.D. of triplicate samples, where ***p>0,001 compared to the untreated group. The experiments were repeated three times, and representative results are shown (**B**).

The NLRP3 inflammasome is activated by a variety of stimuli, including viral, bacterial, fungal and protozoan pathogens. However, the activation of NLRP3 by *T. cruzi* has not been previously reported. To determine the ability of *T. cruzi* to activate NLRP3, we evaluated the secretion of IL-1β by *T. cruzi*-infected peritoneal macrophages (PMs). *T. cruzi* infection induced the secretion of IL-1β by PMs isolated from WT mice, but not from PMs isolated from NLRP3^−/−^ and caspase-1^−/−^ mice (Figure 1A). Lysosomal cathepsins, ROS and/or potassium efflux have been reported to influence NLRP3 activation in response to diverse stimuli [35] [36] [37]. To investigate the role of these molecules in *T. cruzi*-induced NLRP3 activation, PMs from WT mice were infected with *T. cruzi* in the presence of pharmacological inhibitors of cathepsin B (Ca-074ME), NADPH oxidase (apocynin, APO) and potassium channels (glybenclamide, GLB). IL-1β secretion by infected macrophages was inhibited by Ca-074ME in a dose-dependent manner, but was not inhibited by APO or GLB (Figure 1B). Taken together, these results indicate that *T. cruzi* is able to induce NLRP3 activation by a mechanism that involves cathepsin B.

### NLRP3- and caspase-1-deficient mice exhibit high levels of parasitemia and are defective in NO secretion during the acute phase of *T. cruzi* infection

**Figure 2:**
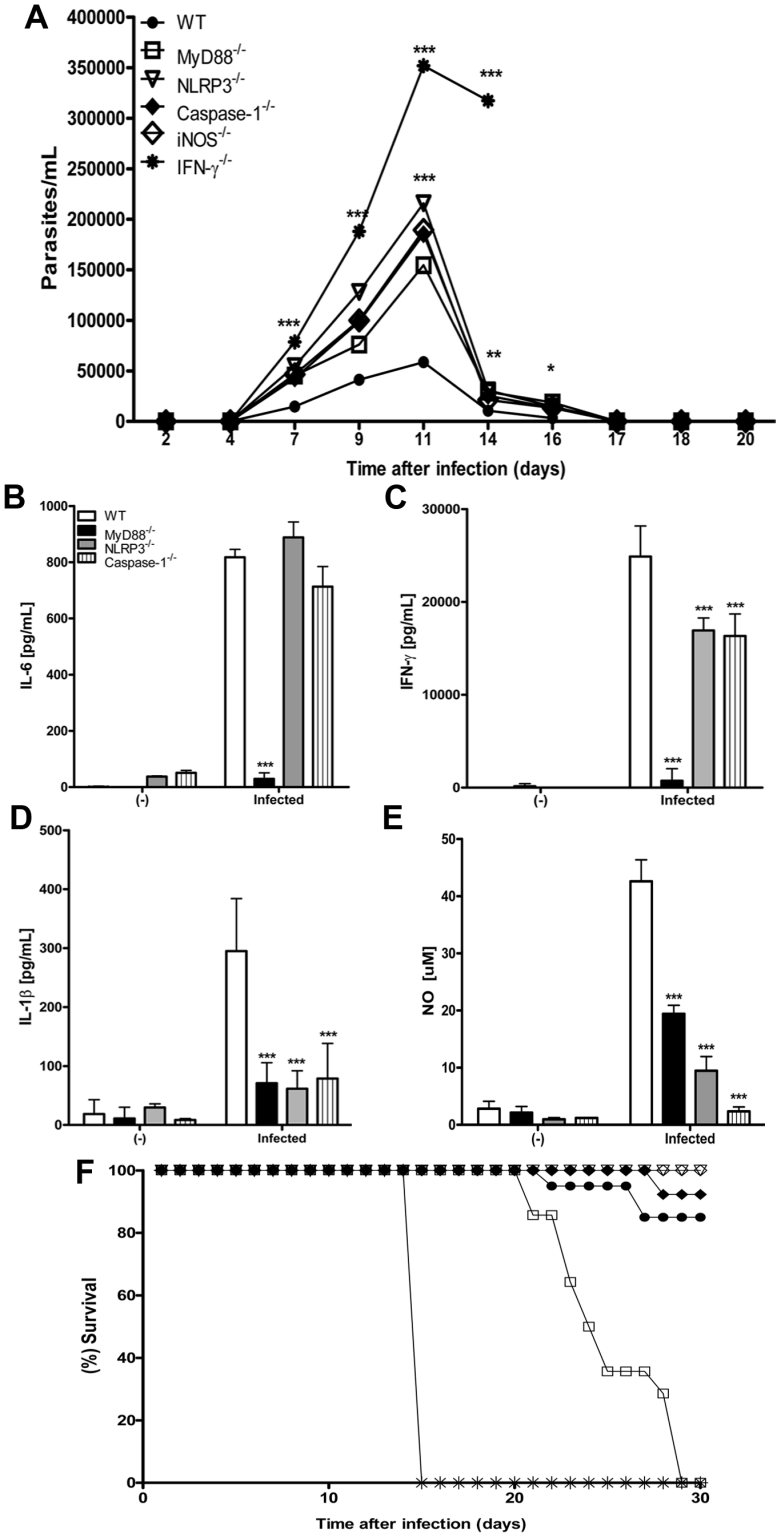
NLRP3 and caspase-1 are involved in the control of *T. cruzi* infection. WT, MyD88^−/−^, NLRP3^−/−^, caspase-1^−/−^, IFN-γ^−/−^ and iNOS^−/−^ mice were subcutaneously infected with 10^3^ *T. cruzi* blood trypomastigotes. Parasitemia was quantified by counting the parasites in 5 µL of tail blood obtained on days 4 to 20 after infection (**A**). Supernatants from cultured spleen cells (3×10^6^ cells/300 µl) isolated from naive (-) or infected (10 days) mice were collected after 24 h. The levels of IL-6 (**B**), IFN-γ (**C**) and IL-1β (**D**) were assessed by ELISA, and NO secretion was assessed by the Griess reaction (**E**). Numbers represent the mean ± S.D. (n = 5). *** p>0,001, ** p>0,01 and * p>0,05 compared to the WT group. Mortality was assessed by daily inspection of the cages (**F**). Experiments were repeated two times with IFN-γ^−/−^ and iNOS^−/−^ mice (n = 6) and five times with MyD88^−/−^, NLRP3^−/−^, caspase-1^−/−^ mice (n = 6) showing similar results.

Next, we evaluated the role of NLRP3 in host resistance to *T. cruzi* infection by assessing parasitemia and mortality in NLRP3^−/−^ and caspase-1^−/−^ mice infected with *T. cruzi* Y. A significantly greater number of *T. cruzi* parasites were found in the blood of both NLRP3^−/−^ and caspase-1^−/−^ mice compared to WT mice (Figure 2A). Interestingly, the degree of parasitemia exhibited by the NLRP3^−/−^ and caspase-1^−/−^ mice was comparable to that exhibited by the MyD88^−/−^ and iNOS^−/−^ mice, which are considered susceptible models for *T. cruzi* infection [18] [19] (Figure 2A and Table S1). The greatest parasite burden was found in IFN-γ*^−/−^* mice (Figure 2A and Table S1). To evaluate the pro-inflammatory responses of NLRP3^−/−^ and caspase-1^−/−^ mice during the acute phase of *T. cruzi* infection, we assessed the level of cytokines and NO in the supernatants of cultured spleen cells isolated 10 days after infection. MyD88^−/−^ mice were used as a positive control for susceptibility to infection. Unlike MyD88^−/−^ spleen cells, the spleen cells isolated from NLRP3^−/−^ and caspase-1^−/−^ mice produced considerable levels of IL-6 (Figure 2B) and IFN-γ (Figure 2C), although they did not produce as much IFN-γ as the WT cells. As expected, IL-1β was produced by splenocytes isolated from WT mice, but not by splenocytes isolated from NLRP3^−/−^, caspase-1^−/−^ and MyD88^−/−^ mice (Figure 2D). In agreement with a previous report [19], the production of NO by spleen cells from infected MyD88^−/−^ mice was significantly reduced compared to spleen cells isolated from WT mice (Figure 2E). Surprisingly, however, the levels of NO produced by splenocytes from NLRP3^−/−^ and especially caspase-1^−/−^ mice were even lower than the levels of NO produced by splenocytes isolated from MyD88^−/−^ mice (Figure 2E). The defect in NO production by spleen cells isolated from the NLRP3^−/−^ and caspase-1^−/−^ mice could explain the high levels of parasitemia observed in the iNOS^−/−^, NLRP3^−/−^ and caspase-1^−/−^ mice (Figure 2A). However, unlike the MyD88^−/−^ and IFN-γ*^−/−^* mice, which succumbed to infection, the NLRP3^−/−^ and caspase-1^−/−^ mice survived infection (Figure 2F). These data suggest that NLRP3 and caspase-1 are required for NO production and parasite control during the acute phase of *T. cruzi* infection.

### NLRP3-dependent NO secretion is critical for *T. cruzi* killing by macrophages

**Figure 3:**
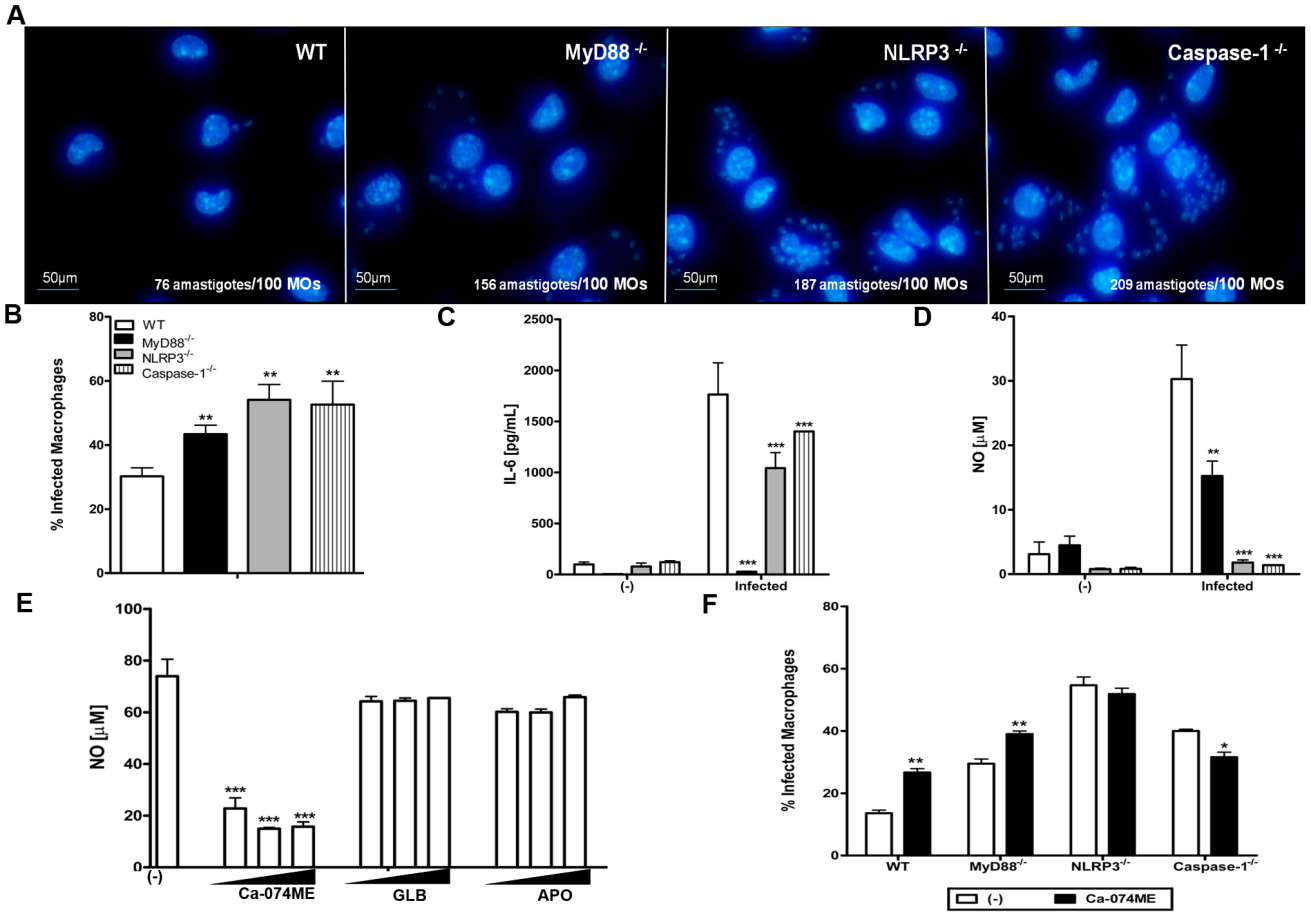
Macrophages isolated from NLRP3^−/−^ and caspase-1^−/−^ mice are impaired in NO secretion and the control of *T. cruzi* infection. PMs (3×10^5^/well of chamber slides) from WT, MyD88^−/−^, NLRP3^−/−^ and caspase-1^−/−^ mice were infected with *T. cruzi* Y trypomastigotes (1∶5) for 2–4 h. Extracellular parasites were removed by washing. After 48 hours, the chambers were fixed with methanol, stained with DAPI and evaluated by fluorescence microscopy (600×). The number of amastigotes (**A**) and the frequency of the infected cells (**B**) were counted in 1000 macrophages from a representative experiment. Experiments were repeated four times with similar results. Supernatants from non-infected (-) and *T. cruzi*-infected PMs (3 parasites per cell) (1,5×10^6^ cells/300 µl) were collected after 24 h to evaluate the IL-6 levels by ELISA (**C**), and after 48 h to measure NO secretion by the Griess reaction (**D**). Numbers represent the mean ± S.D. (n = 5). *** p>0,001 and ** p>0,01 compared to the WT group. *T. cruzi*-infected PMs (3 parasites per cell) (1,5×10^6^ cells/300 µl) from WT mice were treated with Ca-074ME, GLB or APO in doses of 25, 50 and 100 µM, or left untreated. After 48 h, supernatants were collected to evaluate the level of NO production by the Griess reaction. Bars represent the mean ± S.D. of triplicate samples. *** p>0,001 compared to the untreated group. (**E**). PMs (3×10^5^/well of chamber slides) from WT mice were infected with *T. cruzi* Y trypomastigotes (5 parasites/cell) for 2-4 h. After removing extracellular parasites, Ca-074ME (50 µM) was added to the cultures. After 48 h, the chambers were fixed with methanol, stained with DAPI and evaluated by fluorescence microscopy (600×) to determine the frequency of infected cells. Bars represent the mean ± S.D. of triplicate samples, and ** p>0,001 and * p>0,01 compared to the untreated group (**F**).

Because macrophages are the major source of NO during *T. cruzi* infection, the deficiency in NO production exhibited by the caspase-1^−/−^ and NLRP3^−/−^ mice could reflect an impairment in macrophage function. To test this hypothesis, PMs from WT, MyD88^−/−^, NLRP3^−/−^, and caspase-1^−/−^ mice were infected *in vitro* with *T. cruzi* trypomastigotes. After 48 hs, the DAPI-stained slides were analyzed by fluorescence microscopy. The NLRP3^−/−^ and caspase-1^−/−^ macrophages demonstrated a marked deficiency in the control of infection, as the number of amastigotes (Figure 3A) and the frequency of infected cells (Figure 3B) were even higher than in the susceptible MyD88^−/−^ macrophages. Notably, the difference in the amastigotes numbers of the macrophages derived from the *knockouts* and the WT mice was not related to the invasion rate of *T. cruzi*, as similar numbers of amastigotes were observed in the macrophages from WT, NLRP3^−/−^ and caspase-1^−/−^ mice after 2 h of infection (Figure S1A). In contrast, 48 h after infection, the number of amastigotes present in the WT macrophages only was significantly reduced (Figure S1A). Therefore, in the absence of NLRP3 and caspase-1, macrophages were more permissive to parasite replicative or less efficient to kill them, or both. Importantly, pyroptosis (macrophage death induced by inflammasome activation) was not observed during *T. cruzi* infection. The majority of the *T. cruzi*-infected macrophages isolated from WT mice stained with the vital dye acridine orange and did not incorporate ethidium bromide, in contrast to macrophages stimulated with cytosolic flagellin, a potent pyroptotic stimulus [49] (Text S1 and Figure S1B). As observed in the spleen cells from infected mice (Figure 2), unlike MyD88^−/−^ macrophages, macrophages isolated from NLRP3^−/−^ and caspase-1^−/−^ mice secreted the pro-inflammatory cytokine IL-6 (Figure 3C) but did not produce NO (Figure 3D). Furthermore, NLRP3-dependent NO production in response to *T. cruzi* infection appeared to be regulated by cathepsin B, as Ca-074ME, but not APO or GLB, significantly reduced the levels of NO produced by macrophages from WT mice (Figure 3E). These results correlate well with our observations of IL-1β production (Figure 1B). Moreover, treatment with Ca-074ME significantly increased the frequency of infection in macrophages isolated from WT and MyD88^−/−^ mice (Figure 3F), as well as the number of amastigotes found inside these cells (data not shown). In contrast, Ca-074ME treatment had no effect in PMs from NLRP3^−/−^ and even resulted in a small increase in levels of infection in PMs from caspase-1^−/−^ mice (Figure 3F). Taken together, these results suggest that the NLRP3 inflammasome mediates NO production in response to *T. cruzi* infection by a mechanism that involves cathepsin B.

### NLRP3-dependent NO secretion in response to *T. cruzi* infection is mediated by caspase-1 and does not require IL-1R

**Figure 4:**
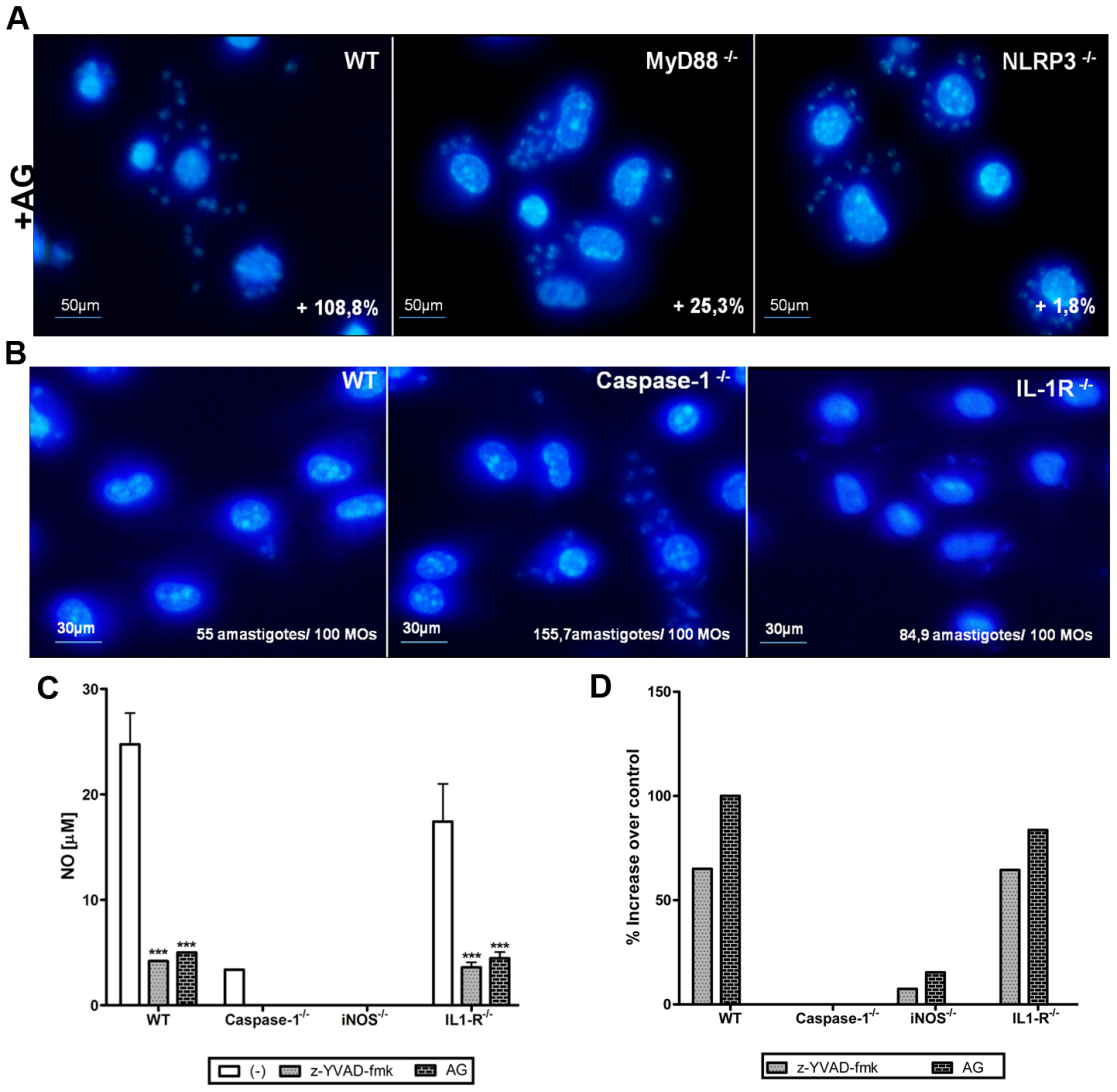
Caspase-1 but not IL-1R mediates *T. cruzi*-induced NO secretion. PMs (3×10^5^/well of chamber slides) from WT, MyD88^−/−^ and NLRP3^−/−^ mice were infected with *T. cruzi* Y trypomastigotes (5 parasites/cell) for 2–4 h. After the extracellular parasites were removed, AG (1 mM) was added to the cultures. After 48 h, the chambers were fixed with methanol, stained with DAPI and evaluated by fluorescence microscopy (600×). The numbers represent the increase in the number of amastigotes compared to untreated cells, and 1000 macrophages were counted from a representative experiment (**A**). PMs (3×10^5^/well of chamber slides) from WT, caspase-1^−/−^ and IL-1R^−/−^ mice (3 parasites/cell) were infected with *T. cruzi* Y trypomastigotes (5 parasites/cell) for 2–4 h. Chambers were obtained as described and evaluated by fluorescence microscopy (600×). The number of amastigotes was counted in 1000 macrophages from a representative experiment. The experiments were repeated two times with similar results (**B**). *T. cruzi*-infected PMs (3 parasites per cell) (1,5×10^6^ cells/300 µl) from WT, caspase-1^−/−^, iNOS^−/−^ and IL-1R^−/−^ mice were treated or not with z-YVAD-fmk (10 µM) or AG (1 mM). After 48 h, nitrite accumulation was measured in the culture supernatants by the Griess reaction. Bars represent the mean ± S.D. of triplicate samples. *** p>0,001 compared to the untreated group (**C**). *T. cruzi*-infected PMs (3×10^5^/well of chamber slides) from WT, caspase-1^−/−^, iNOS^−/−^ and IL-1R^−/−^ mice (3 parasites/cell) were treated or not with z-YVAD-fmk (10 µM) or AG (1 mM). Chambers were obtained as described above and evaluated by fluorescence microscopy (600×). The frequency of infected macrophages was counted in 1000 macrophages from a representative experiment. Bars represent the rate of increase compared to untreated cells, where *** p>0,001 compared to the untreated group. The experiments were repeated two times with similar results (**D**).

To confirm the role of NLRP3-dependent NO secretion in the control of *T. cruzi* infection, macrophages were infected in the presence of AG, a selective iNOS inhibitor. Treatment with AG increased the number of intracellular parasites found in WT and MyD88^−/−^ macrophages by 100% and 25%, respectively (Figure 4A), indicating that the NO produced by these macrophages contributes to the control of *T. cruzi* infection. Conversely, no significant effect was observed in NLRP3^−/−^ macrophages treated with AG (Figure 4A), supporting the idea that NLRP3-mediated NO secretion is involved in the control of parasite replication. Because NLRP3 activation leads to caspase-1 activation and consequent IL-1β secretion, we next asked whether caspase-1 and IL-1β are required for NLRP3-dependent NO production in response to *T. cruzi* infection, and what role these molecules play in the control of infection by macrophages. In contrast to the caspase-1^−/−^ macrophages, we observed only a minor increase in the number of amastigotes in macrophages isolated from IL-1R^−/−^ mice compared to macrophages isolated from WT mice (Figure 4B). These results correlate with the ability of the IL-1R^−/−^ macrophages to produce NO (Figure 4C; white bars). Moreover, in the presence of the caspase-1 inhibitor (z-YVAD-fmk) or AG, the NO production (Figure 4C) and the capacity to control of *T. cruzi* infection (Figure 4D) of the WT and IL-1R^−/−^ macrophages were abrogated. Importantly, treatment with z-YVAD-fmk or AG had no significant effect on NO production (Figure 4C) and amastigote numbers (Figure 4D) in macrophages from caspase-1^−/−^ and iNOS^−/−^ mice. This finding indicates that caspase-1 plays a major role in the induction of NLRP3-dependent NO production and the control of *T. cruzi* infection by macrophages.

### NLRP3-mediated NO production acts synergistically with MyD88-mediated pathways to control *T. cruzi* infection

**Figure 5:**
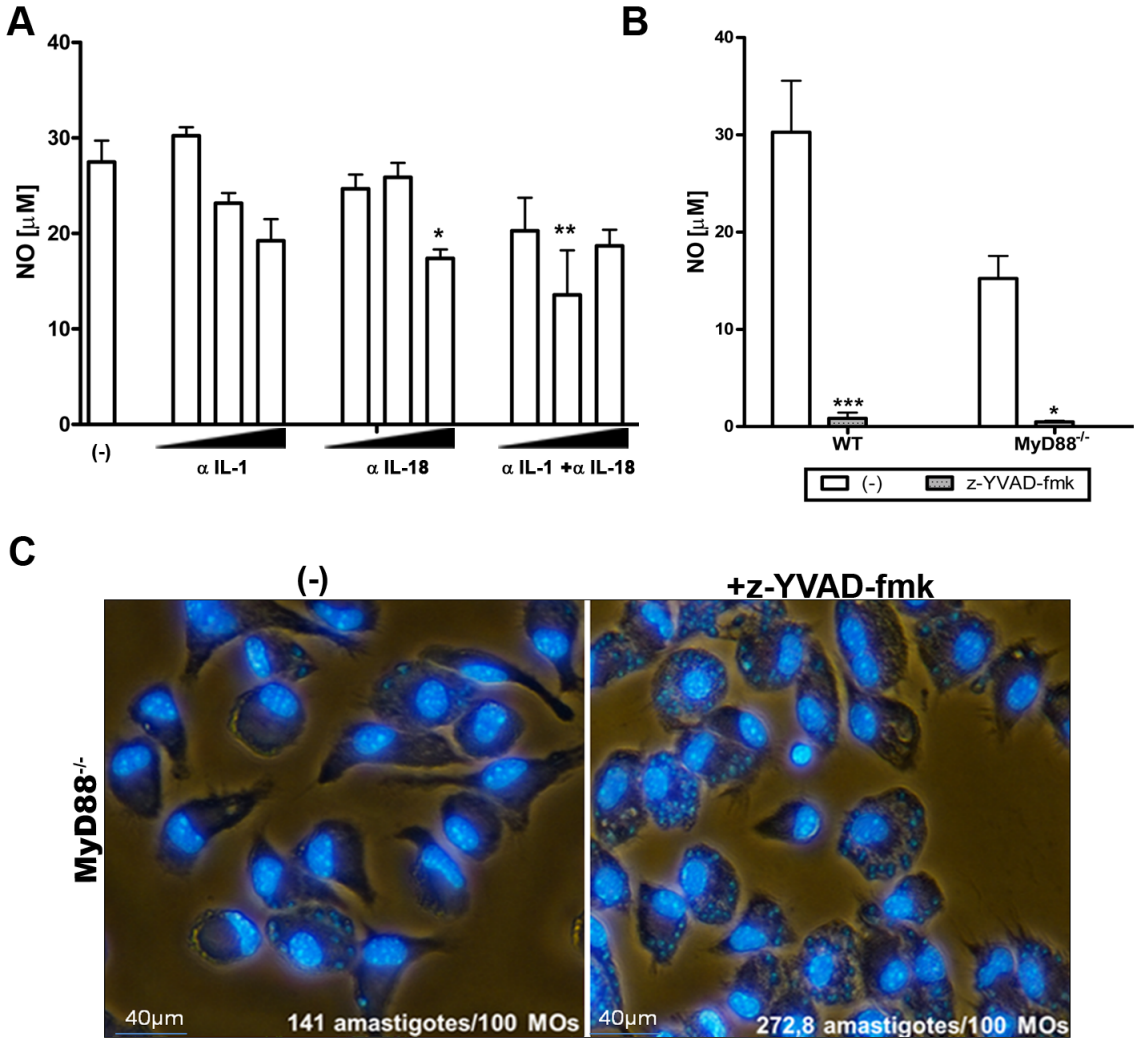
NLRP3-mediated NO production acts synergistically with MyD88-mediated pathways to control *T. cruzi* infection. PMs from WT mice were infected with *T. cruzi* Y (3 parasites per cell) (1,5×10^6^ cells/300 µl) in the presence of neutralizing antibodies against IL-1β, IL-18 or both cytokines in doses of 0,1, 1 and 10 µg/mL, or left untreated. After 48 h, nitrite accumulation was measured in the culture supernatants by the Griess reaction. Bars represent the mean ± S.D. of triplicate samples where ** p>0,01 and * p>0,05 when compared to untreated group (**A**). *T. cruzi*-infected PMs from WT and MyD88^−/−^ mice (3 parasites/cell) were treated or not with z-YVAD-fmk (10 µM). After 48 h, nitrite accumulation was measured in culture supernatants by Griess Rection. Bars represent the mean ± S.D. of triplicate samples where *** p>0,001 and * p>0,01 when compared to untreated group (**B**). PMs (3×10^5^/well of chamber slides) from MyD88^−/−^ mice were infected with tripomastigotes from *T. cruzi* Y (5 parasites/cell) for 2–4 h. After removal of extracellular parasites, z-YVAD-fmk (10 µM) was added to cultures. After 48 h chambers were prepared and analyzed as described above to evaluate the numbers of amastigotes. Results were obtained in 1000 counted macrophages from a representative experiment Experiments were repeated three times with the same profile of results (**C**).

As demonstrated in Figure 3D, MyD88^−/−^ macrophages secreted NO, although at reduced levels compared to WT macrophages. Because MyD88 participates in the IL-1R and IL-18R signaling pathways, these results confirm that NO production in response to *T. cruzi* infection is mediated by a signaling pathway that involves NLRP3/caspase-1 and is independent of IL-1β and IL-18. In fact, the neutralization of IL-18 or IL-1β plus IL-18 reduced NO production by *T.* cruzi-infected macrophages from WT mice by only 15–20% (Figure 5A). Notably, treatment with z-YVAD-fmk abrogated the secretion of NO by MyD88^−/−^ macrophages (Figure 5B) and rendered them highly susceptible to *T. cruzi* replication (Figure 5C). Together, these results suggest that MyD88 and caspase-1, as well as IL-1β and IL-18 to a lesser extent, cooperate to clear *T. cruzi* infection by inducing NO production.

## Discussion

**Figure 6:**
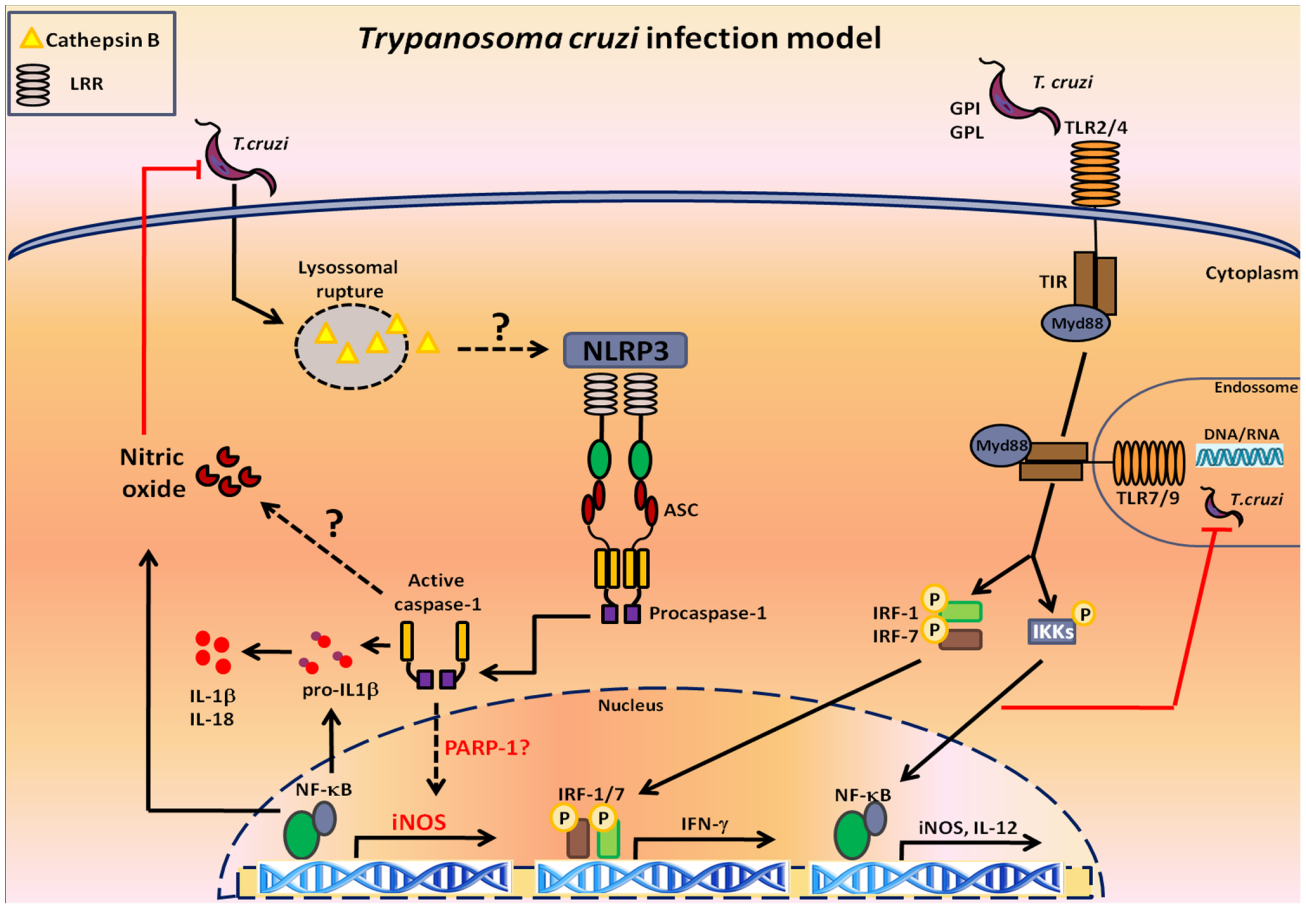
The proposed model for NLRP3-mediated control of *T. cruzi*. TLR2, TLR4, TLR7 and TLR9 recognize *T. cruzi* PAMPs and control infection by MyD88 adaptor molecule-mediated effector mechanisms. *T. cruzi* infection leads to lysosomal disruption, which may result in cathepsin B release into the cytosol and NLRP3 inflammasome activation. The activation of the NLRP3 inflammasome results in caspase-1 activation, which induces NO secretion in an IL-1R-independent manner. Caspase-1-dependent NO secretion appears to cooperate with MyD88-dependent pathways to control *T. cruzi* infection.

Cells from the innate immune system play a key role in sensing and controlling the acute phase of *T. cruzi* infection. While TLR2, TLR4, TLR7, TLR9 and NOD1 play well-established roles in host resistance to *T. cruzi* infection, it is unknown whether inflammasomes, multiprotein platforms that are emerging as central regulators in many infections and inflammatory pathologies, are involved. Herein, we show that *T. cruzi* activates NLRP3 inflammasomes by a mechanism that involves lysosomal cathepsins. NLRP3 and the effector protease caspase-1 appear to participate in controlling the acute phase of *T. cruzi* infection, as NLRP3^−/−^ and caspase-1^−/−^ mice exhibit a very prominent peak parasitemia, despite their ability to secrete IL-6 and IFN-γ. However, in the absence of NLRP3 and caspase-1, NO production is significantly impaired, indicating the involvement of NLRP3 inflammasomes in inducing NO secretion. The deficiency in NO secretion reflects in the inability of macrophages to control intracellular amastigotes. Interestingly, NLRP3-dependent NO secretion is completely abrogated in the absence of caspase-1, but not in the absence of IL-1R, which could explain the ability of IL-1R^−/−^ macrophages, but not caspase-1^−/−^ macrophages, to prevent *T. cruzi* replication. In fact, macrophages isolated from MyD88^−/−^ mice, which are unable to respond to TLRs agonists (except TLR3), IL-1 and IL-18, secrete NO, although at low levels compared to WT cells. Moreover, the inhibition of caspase-1 in MyD88^−/−^ macrophages renders them susceptible to *T. cruzi* infection as NLRP3^−/−^ and caspase-1^−/−^ macrophages. Collectively, our data demonstrate the existence of a novel NO induction pathway that involves NLRP3 inflammasomes (Figure 6).

Recently, it has been reported that common pathways involving the production of ROS, disruption of lysosomes and/or potassium efflux influence NLRP3 activation, as they are activated by several PAMPs and DAMPs that are not structurally related [39] [40] [41]. *T. cruzi* infection induced IL-1β secretion by spleen cells as well as by macrophages isolated from WT mice, but not by cells isolated from NLRP3^−/−^ and caspase-1^−/−^ mice, which suggests that *T. cruzi* activates NLRP3 inflammasomes. Importantly, IL-1β production was also reduced in MyD88^−/−^ cells, which is expected since pro-IL-1β is transcriptionally regulated by signals dependent on TLR [50]. Interestingly, the pharmacological inhibition of cathepsin B abrogated *T. cruzi*-induced IL-1β secretion, suggesting that lysosomal pathways are involved in NLRP3 activation in response to *T. cruzi* infection. In fact, *T. cruzi* invades a variety of cell types by recruiting lysosomes to the plasma membrane, which they fuse with to form a steady parasitophorous vacuole [51]. *T. cruzi* requires the acidic environment of the lysosome to exit the parasitophorous vacuole and replicate in cell cytosol. We suggest that the lysosomes are disrupted by this process, leading to liberation of cathepsins which then mediate NLRP3 activation by an as yet unknown mechanism.

NLRP3 inflammasomes appear to control *T. cruzi* replication during the acute phase of infection, as NLRP3^−/−^ and caspase-1^−/−^ mice exhibit a very prominent peak parasitemia 11 d after infection. While NLRP3 is involved in many inflammatory disorders, little is known about the role of NLRP3 in controlling infections. Recently, we demonstrated that NLRP3 and AIM2 inflammasomes are involved in the control of *Brucella abortus* infection through an IL-1β- and IL-18-dependent mechanism [45]. NLRP3-induced IL-1β secretion also mediates host resistance to *C. rodentium*, [43], *M. kansasii* [42], *S. typhimurium* [44], Influenza A virus [46] and *C. albicans* [32] [52]. However, NLRP3 does not appear to play a role in the control of the protozoan parasite *Plasmodium* [33] [53]. Conversely, the absence of NLRP3 or IL-1β resulted in increased survival of the malaria strain *P. chabaudi adami* DS, indicating that NLRP3 promotes malaria pathology. The first demonstration of the involvement of inflammasomes in the control of a protozoan infection was recently described in collaboration with our group [54]. In this article, we demonstrated that the NLRP3 inflammasome is involved in the control of *L. amazonensis*, *L. braziliensis* and *L. infantum chagasi* through a mechanism that involves IL-1R-dependent NO production.

Interestingly, deficiencies in NLRP3 and caspase-1 result in high levels of parasites in the blood and inside macrophages, which is similar to results from MyD88^−/−^ and iNOS^−/−^ mice. However, iNOS^−/−^, NLRP3^−/−^ and caspase-1^−/−^ mice do not succumb to infection, unlike MyD88^−/−^ mice. The pronounced deficiency in IFN-γ production exhibited by MyD88^−/−^ cells, but not by NLRP3^−/−^ and caspase-1^−/−^ cells, could explain this difference, as IFN-γ*^−/−^* mice also succumb early to infection. It is well-established that the deficiency in IFN-γ secretion by MyD88^−/−^ mice infected with *T. cruzi* reflects an impairment, not only in the microbicidal capacity of the macrophages but also in DC and TCD8 cell functions, as well an antibody switch to IgG2a and FCγR induction [55]. This could also explain why the highest number of parasites was observed in IFN-γ*^−/−^* mice compared to all the other immunodeficient mouse strains studied here.

Despite the ability to secrete pro-inflammatory cytokines, spleen cells and macrophages from NLRP3*^−/−^* and caspase-1*^−/−^* mice were not able to secrete NO in response to *T. cruzi* infection. The role of NO in the control of *T. cruzi* infection is well established [56]. Mice that are deficient in iNOS [18] or have been treated with pharmacological iNOS inhibitors [57] [58] exhibit high levels of parasites in the blood and can succumb to the acute phase of infection, depending on the parasite and mouse strain. In our model, the NLRP3^−/−^ and caspase-1^−/−^ mice behaved in a manner identical to the iNOS^−/−^ mice, with a higher magnitude of peak parasitemia compared to WT mice; however, most of the NLRP3^−/−^, caspase-1^−/−^ and iNOS^−/−^ mice survived infection. Macrophages isolated from the NLRP3^−/−^ and caspase-1^−/−^ mice failed to kill amastigotes and exhibited an infection index that was even higher than that observed in MyD88^−/−^ macrophages. The incapacity to control *T. cruzi* infection demonstrated by NLRP3^−/−^ and caspase-1^−/−^ macrophages correlated with a deficiency in NO secretion. Moreover, adding AG to *T. cruzi*-infected macrophages increased the parasite burden in WT and MyD88^−/−^ macrophages. Conversely, no effect of AG was observed in NLRP3^−/−^ cells, indicating that this inflammasome is required for NO production in response to *T. cruzi* infection.

The maturation and secretion of the pro-inflammatory cytokines IL-1β and IL-18 and the induction of pyroptosis, a specialized form of inflammatory cell death, are the major effector mechanisms induced by activated caspase-1 [21] [23]. While pyroptosis is known to pay a role in controlling bacterial infections [59], this specialized cell death process did not appear to occur during *T. cruzi* infection, as the majority of the macrophages were viable 48 hs after infection. Recently, we described a caspase-1-dependent pathway for iNOS activation in response to cytosolic flagellin that is mediated by NAIP5/NLRC4-inflammasomes [60]. Caspase-1-dependent iNOS activation is involved in the control of *Legionella pneumophilla* [60] and *Salmonella typhimurium* (unpublished data from our group) by macrophages. Interestingly, MyD88, IL-1β and IL-18 are dispensable for iNOS activation in response to cytosolic flagellin. IL-1β and IL-18 also appear to play a minor role in the induction of NO production and the capacity of macrophages to control *T. cruzi* infection, as a genetic defect in IL-1R, as well as the neutralization of IL-1β and IL-18, have little influence on these activities. Conversely, NO secretion was not observed in caspase-1^−/−^ macrophages or in macrophages treated with z-YVAD-fmk, confirming the existence of a pathway of NLRP3-mediated NO secretion in response to *T. cruzi* infection that is dependent of caspase-1 but independent of IL-1β and IL-18, the best studied caspase-1 substrates. However, the mechanism by which caspase-1 mediates NO secretion remains unclear. Recently, it was demonstrated that a chromatin-associated multifunctional enzyme PARP1 (also called ARTD1) negatively regulates the transcription of NF-κB-dependent target genes by a pathway that requires NLRP3/caspase-1/caspase-7 activation [61]. Mutating the PARP1 cleavage site D214 rendered PARP-1 uncleavable and inhibited PARP1 release from chromatin and chromatin decondensation, thereby inhibiting the expression of cleavage-dependent NF-κB target genes such as *il-6*, *cfs2* and *lif*, but not *ip-10* [61]. Earlier studies showed that PARP1 cleavage is essential for stimulus-dependent transcriptional activation of transiently transfected reporter plasmids containing the *inos* and *mip2* NF-κB sites in mouse lung fibroblasts (MLFs) [62] [63]. Additionally, *inos* expression is significantly reduced in the presence of noncleavable PARP1 (D214N), compared to cleavable PARP1 (WT or enzymatically inactive) [64]. It was recently demonstrated that treatment with Olaparib (a pharmacological inhibitor of PARP-1 enzymatic activity) reduced *T. cruzi* replication in VERO cells [65]. However, the enzymatic activity of PARP-1 is not related to the role of cleaved PARP-1 in the regulation of chromatin condensation and access to transcription factors [60]. Therefore, further studies are required to understand the molecular machinery involved in the regulation of iNOS activation by the caspase-1/PARP-1 axis.

TLR pathways [66], IFN-γ [67] and IFN-γ-related cytokines such as IL-12 [15] [68] are known to be the major inducers of NO production in response to *T. cruzi* infection. However, we demonstrate here that pharmacological inhibition of caspase-1 abrogates NO secretion by spleen cells and macrophages from WT and MyD88^−/−^ mice and renders their macrophages as susceptible to *T. cruzi* infection as NLRP3^−/−^, caspase-1^−/−^ and iNOS^−/−^ cells. This indicates that the requirement of NLRP3 inflammasomes for NO secretion bypasses the need for MyD88, and identifies a novel NLRP3-dependent pathway that stimulates NO production in response to *T. cruzi* infection. Despite the minor effect of IL-1R and IL-18 on NO secretion in response to *T. cruzi* infection, the amount of NO produced by macrophages and cultured spleen cells from MyD88^−/−^ mice is significantly lower than that produced by WT cultures. The fact that caspase-1 inhibition in MyD88^−/−^ cells abrogates NO production and renders macrophages more susceptible to *T. cruzi* infection suggests that the NLRP3 and MyD88 pathways cooperate to induce NO secretion and promote host resistance to *T. cruzi*. Taken together, our data describe a novel effector mechanism mediated by NLRP3 inflammasomes that contributes to the control of *T. cruzi* infection.

## Supporting Information

Figure S1 **Inflammatory cell death is not involved in the control of** ***T. cruzi*** **by macrophages.** PMs (3×105/well of chamber slides) from WT mice were infected with *T. cruzi* Y trypomastigotes (1∶5). After 2 h or 48 h, the chambers were fixed with methanol, stained with DAPI and evaluated by fluorescence microscopy (600×). Bars represent the mean ± S.D. of triplicate samples of the number of amastigotes found in 100 macrophages (MOs). * p>0,01 compared to the 2 h group. The number of amastigotes was counted in 1000 PMs from a representative of two experiments (**A**). PMs (3×105/well of chamber slides) from WT mice were stimulated with 6 µg/ml of purified flagellin from *Bacillus subtilis* inserted into DOTAP (Fladot) for 6 h, or were infected with *T. cruzi* Y (5∶1) for 6–48 h. Cytotoxicity was assessed by fluorescence microscopy according to the loss of vital acridine orange staining and incorporation of ethidium bromide (**B**).
(TIF)

Table S1 **Effect of NLRP3 and caspase-1 in the control of the acute phase of** ***T. cruzi*** **infection.** WT, MyD88^−/−^, NLRP3^−/−^, Caspase-1^−/−^, IFN-γ^−/−^ and iNOS^−/−^ mice were subcutaneously infected with 10^3^ *T. cruzi* blood trypomastigotes. Parasitemia was quantified by counting the parasites in 5 µL of tail blood obtained on days 4 to 20 after infection. Global Parasitemia (GP) represents the mean of sum of total blood parasites found in each mouse strain and S.D. (n = 6). *** p>0,001 compared to the WT group and #p>0,001 compared to IFN-γ^−/−^ group. No significant difference was observed among MyD88^−/−^, iNOS^−/−^, Caspase-1^−/−^ and NLRP3^−/−^ groups. AUC – Area under Curve. Experiments were repeated two times with IFN-γ^−/−^ and iNOS^−/−^ mice and five times with MyD88^−/−^, NLRP3^−/−^, caspase-1^−/−^ mice showing similar results.
(DOCX)

Text S1 **Ethidium bromide and acridine orange stain.**
(DOCX)
