# Supplementary figures and images for: NLRP3 Controls Trypanosoma cruzi Infection through a Caspase-1-Dependent IL-1R-Independent NO Production
Source: PLoS Negl Trop Dis. 2013 Oct 3;7(10):e2469. doi: 10.1371/journal.pntd.0002469 (PMC3789781; doi:10.1371/journal.pntd.0002469)

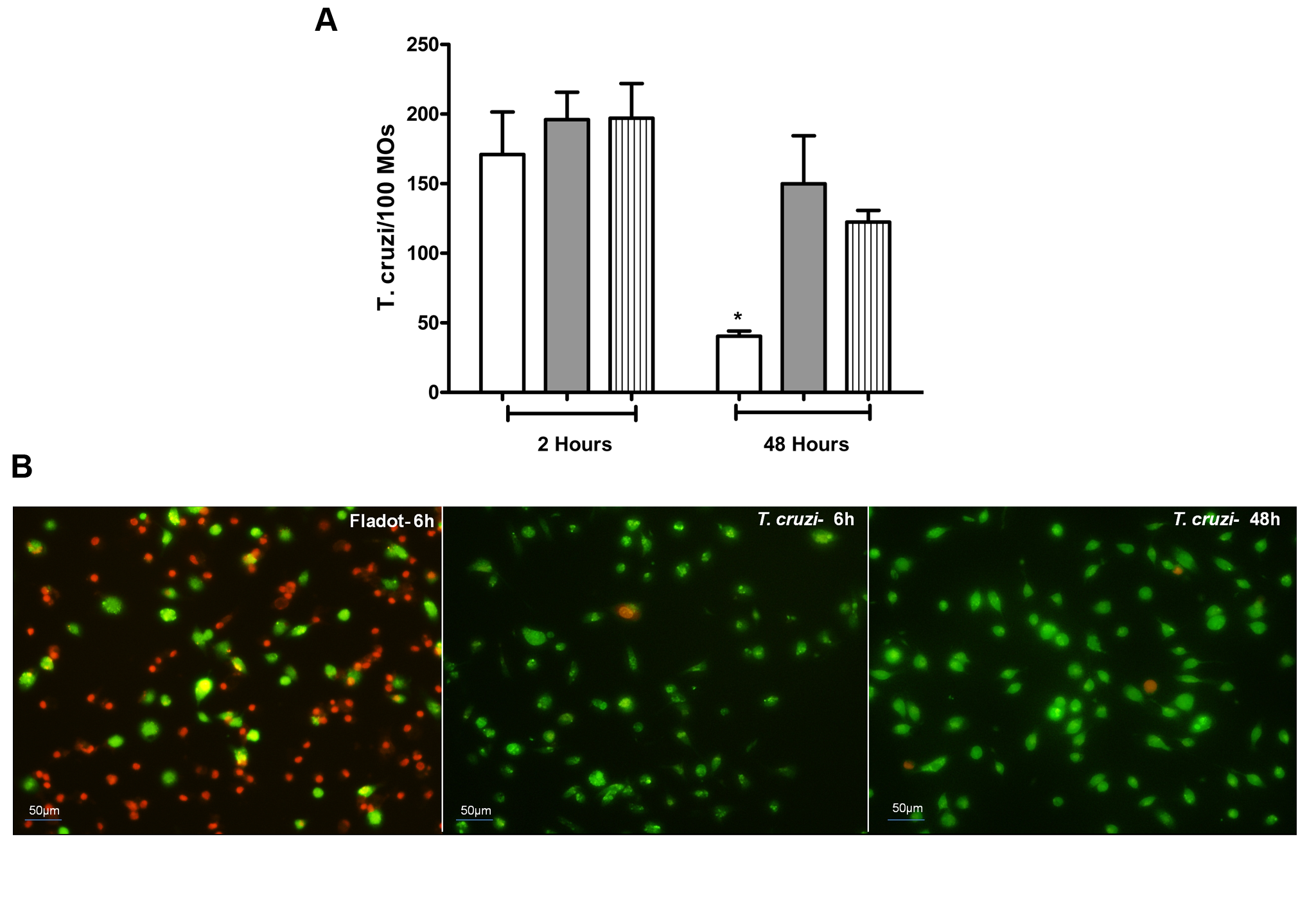

Supplement: Figure S1 — Inflammatory cell death is not involved in the control of T. cruzi by macrophages. PMs (3×10^5^/well of chamber slides) from WT mice were infected with T. cruzi Y trypomastigotes (1∶5). After 2 h or 48 h, the chambers were fixed with methanol, stained with DAPI and evaluated by fluorescence microscopy (600×). Bars represent the mean ± S.D. of triplicate samples of the number of amastigotes found in 100 macrophages (MOs). * p>0,01 compared to the 2 h group. The number of amastigotes was counted in 1000 PMs from a representative of two experiments (A). PMs (3×10^5^/well of chamber slides) from WT mice were stimulated with 6 µg/ml of purified flagellin from Bacillus subtilis inserted into DOTAP (Fladot) for 6 h, or were infected with T. cruzi Y (5∶1) for 6–48 h. Cytotoxicity was assessed by fluorescence microscopy according to the loss of vital acridine orange staining and incorporation of ethidium bromide (B). (TIF) [file pntd.0002469.s001.tif]
