# Supplementary material for: NLRP3 Controls Trypanosoma cruzi Infection through a Caspase-1-Dependent IL-1R-Independent NO Production
Source: PLoS Negl Trop Dis. 2013 Oct 3;7(10):e2469. doi: 10.1371/journal.pntd.0002469 (PMC3789781; doi:10.1371/journal.pntd.0002469)
Supplement: Table S1 — Effect of NLRP3 and caspase-1 in the control of the acute phase of T. cruzi infection. WT, MyD88−/−, NLRP3−/−, Caspase-1−/−, IFN-γ−/− and iNOS−/− mice were subcutaneously infected with 103 T. cruzi blood trypomastigotes. Parasitemia was quantified by counting the parasites in 5 µL of tail blood obtained on days 4 to 20 after infection. Global Parasitemia (GP) represents the mean of sum of total blood parasites found in each mouse strain and S.D. (n = 6). *** p>0,001 compared to the WT group and #p>0,001 compared to IFN-γ−/− group. No significant difference was observed among MyD88−/−, iNOS−/−, Caspase-1−/− and NLRP3−/− groups. AUC – Area under Curve. Experiments were repeated two times with IFN-γ−/− and iNOS−/− mice and five times with MyD88−/−, NLRP3−/−, caspase-1−/− mice showing similar results. (DOCX) [file pntd.0002469.s002.docx]

Supplemental Table I - Global Parasitemia

| **STRAINS** | **GP** | **SD** | **AUC** |
| --- | --- | --- | --- |
| C57BL/6 | 1.30E+05^#^ | 1.40E+04 | 297467 |
| MyD88^-/-^ | 3.30E+05^*#^ | 1.94E+04 | 753333 |
| Caspase-1^-/-^ | 3.60E+05^*#^ | 3.73E+04 | 850667 |
| NLRP3^-/-^ | 4.04E+05^*#^ | 2.88E+04 | 931000 |
| iNOS^-/-^ | 3.70E+05^*#^ | 1.57E+04 | 863333 |
| IFN-γ^-/-^ | 9.40E+05^*^ | 6.36E+04 | 2405000 |

**Effect of NLRP3 and caspase-1 in the control of acute phase of *T. cruzi* infection**. WT, MyD88^-/-^, NLRP3^-/-^, Caspase-1^-/-^, IFN-γ^-/-^ and iNOS^-/-^ mice were subcutaneously infected with 10^3^ *T. cruzi* blood trypomastigotes. Parasitemia was quantified by counting the parasites in 5μL of tail blood obtained on days 4 to 20 after infection. Global Parasitemia (GP) represents the mean of sum of total blood parasites found in each mouse strain and S.D. (n=6). *** p>0,001 compared to the WT group and #p>0,001 compared to IFN-γ^-/-^ group. No significant difference was observed among MyD88^-/-^, iNOS^-/-^, Caspase-1^-/-^ and NLRP3^-/-^ groups. AUC – Area under Curve. Experiments were repeated two times with IFN-γ^-/-^ and iNOS^-/-^ mice and five times with MyD88^-/-^, NLRP3^-/-^, caspase-1^-/-^ mice showing similar results.
