# Supplementary material for: NLRP3 Controls Trypanosoma cruzi Infection through a Caspase-1-Dependent IL-1R-Independent NO Production
Source: PLoS Negl Trop Dis. 2013 Oct 3;7(10):e2469. doi: 10.1371/journal.pntd.0002469 (PMC3789781; doi:10.1371/journal.pntd.0002469)
Supplement: Text S1 — Ethidium bromide and acridine orange stain. (DOCX) [file pntd.0002469.s003.docx]

**Text S1**

**Ethidium bromide and acridine orange stain**

Death of PMs stimulated with purified flagellin from *B. subtillis* inserted into DOTAP (3μl/ml) (49) or infected with trypomastigotes from *T. cruzi* (1:5) was assessed using ethidium bromide (EtBr) incorporation in combination with acridine orange (AO) (Sigma) staining as described previously (49). Briefly, a solution of PBS containing EtBr and AO (50 μg/mL v/v) was added to cells after 1,5-48 h. Images were acquired using an inverted fluorescence microscope with original magnification of 400x.
